# Key Factors for Thymic Function and Development

**DOI:** 10.3389/fimmu.2022.926516

**Published:** 2022-06-30

**Authors:** Valentin P. Shichkin, Mariastefania Antica

**Affiliations:** ^1^ OmniFarma, Kyiv, Ukraine; ^2^ Rudjer Boskovic Institute, Zagreb, Croatia

**Keywords:** thymus, thymic epithelial cells (TEC), thymic microenvironment, thymus regeneration, T cells, intrathymic regulators, thymic stem cells

## Abstract

The thymus is the organ responsible for T cell development and the formation of the adaptive immunity function. Its multicellular environment consists mainly of the different stromal cells and maturing T lymphocytes. Thymus-specific progenitors of epithelial, mesenchymal, and lymphoid cells with stem cell properties represent only minor populations. The thymic stromal structure predominantly determines the function of the thymus. The stromal components, mostly epithelial and mesenchymal cells, form this specialized area. They support the consistent developmental program of functionally distinct conventional T cell subpopulations. These include the MHC restricted single positive CD4^+^ CD8^-^ and CD4^-^ CD8^+^ cells, regulatory T lymphocytes (Foxp3^+^), innate natural killer T cells (iNKT), and γδT cells. Several physiological causes comprising stress and aging and medical treatments such as thymectomy and chemo/radiotherapy can harm the thymus function. The present review summarizes our knowledge of the development and function of the thymus with a focus on thymic epithelial cells as well as other stromal components and the signaling and transcriptional pathways underlying the thymic cell interaction. These critical thymus components are significant for T cell differentiation and restoring the thymic function after damage to reach the therapeutic benefits.

**Graphical Abstract:**
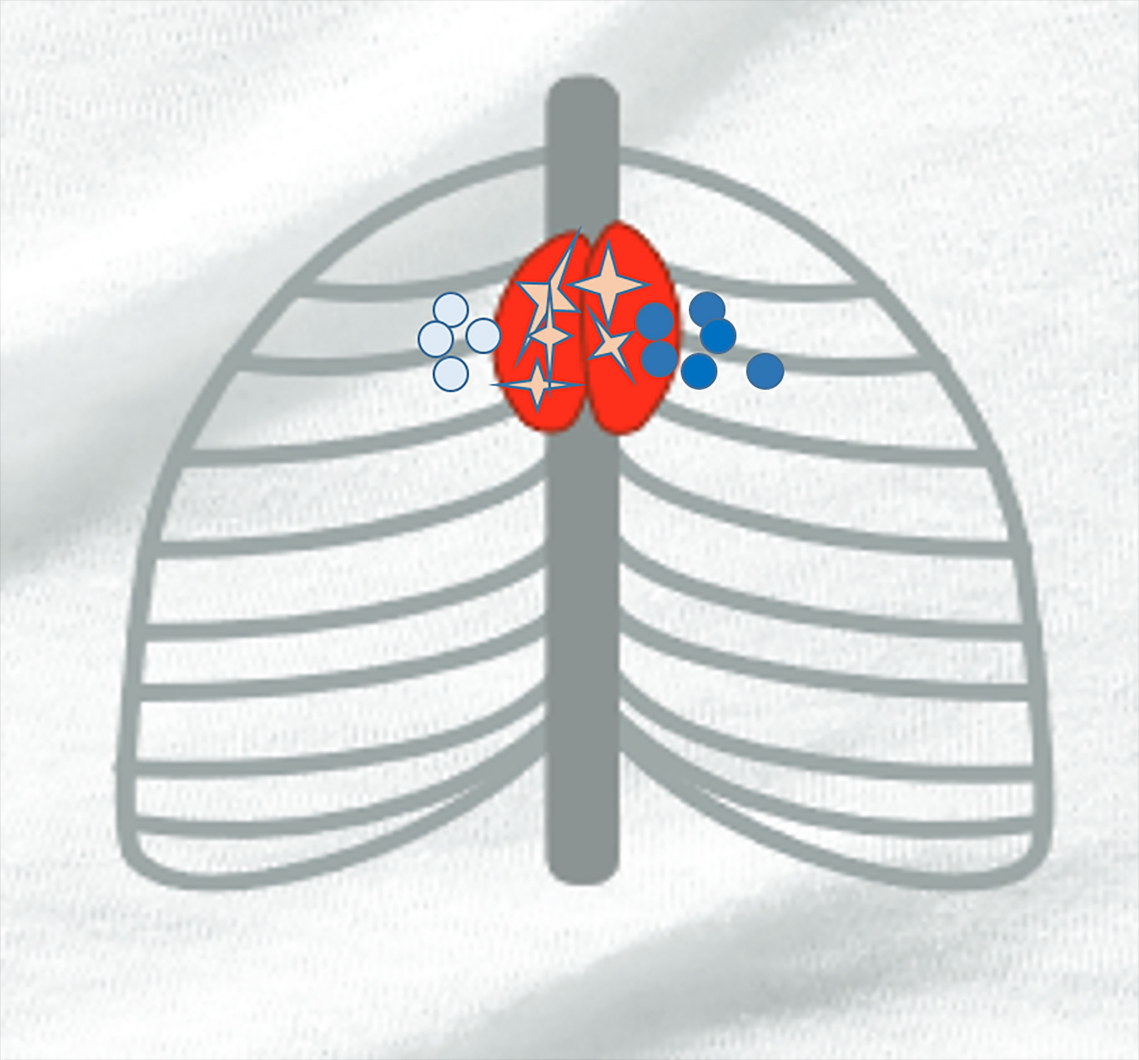


## Introduction

**Figure 1:**
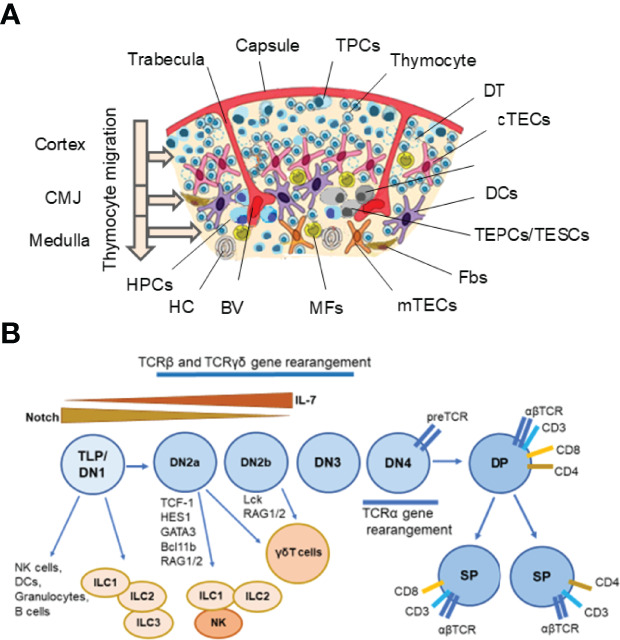
Thymus cell architecture **(A)**, and T cell and innate lymphoid cell (ILC) development in the thymus **(B)**. The thymus consists of two lobes that are separated by connective tissue strands (trabeculae) in lobules. Each thymic lobule consisted of the cortex and medulla. The cortex contains CD34^+^ uncommitted pluripotent hematopoietic precursor cells (HPCs) entering the thymus at the cortico-medullary junction (CMJ) and migrating to the capsule, committed double negative (DN) CD4^−^CD8^−^ T precursor cells (TPCs) located in the subcapsular region (DN1–DN4 stages), and immature double positive (DP) CD4^+^CD8^+^ (Pre-DP) cortical thymocytes migrating through the cortex and CMJ to the medullar zone. The medulla contains single positive (SP) CD4^+^ and CD8^+^ naïve thymocytes migrating to the periphery after maturing. Stromal-epithelial compartment of the thymus is submitted by minor populations of EpCam^+^ (CD326^+^) Foxn1^+^ bipotent thymic epithelial precursor cells/thymic epithelial stem cells (TEPCs/TESCs), and mesenchymal stem cells (MSCs) located probably into the thymic parenchyma close to the CMJ region, as well as EpCam^+^CD205^+^ cortical thymic epithelial cells (cTECs) located in the cortex and EpCam^+^Air^+^ medullary thymic epithelial cells (mTECs) located in the medulla. The cortex and medulla also contain macrophages (MFs), fibroblasts (Fbs), and dendritic cells (DCs) that, together with cTECs and mTECs, participate in the differentiation, maturation, and positive and negative selection of thymocytes. T cell and ILC lineages diverge at the stages of early T precursors/double negative 1 (ETP/DN1) and the DN2-DN3 transition stage. Depending on the status of the TCR loci, the strength of Notch signaling and activities of E-ld proteins and Bcl11b, multipotent TLPs may develop conventional αβ T cells or acquire innate-like properties and give rise to thymic natural killer (NK) cells, DCs, granulocytes, B cells, one of three ILC subsets and invariant γδ T cells. Resident ILC progenitors have been suggested to originate from failed T cell development and locally maintain the mature ILC pool. BV, Blood Vessel; DT, Dead Thymocytes; HC, Hassall’s Corpuscle. **(A)** modified from Shichkin and Antica, 2020 (9); the article is licensed under a Creative Commons Attribution 4.0 International License. **(B)** modified from Shin and McNagny, 2021 (138); the article is distributed under the terms of the Creative Commons Attribution License (CC BY).

The thymus controls the constant production of self-tolerant T lymphocytes throughout the whole life of the organism. This lymphoid organ consists of two lobes, each enveloped by connective tissue. The outer compartment of the lobes is the cortex, where early-stage thymocytes develop. The medulla is the inner compartment, where later thymocyte stages develop. The intersection of these regions is the cortical-medullary junction (CMJ), where blood vessels transport the hematopoietic progenitors from the bone marrow to the thymus and mature T lymphocytes from the thymus to the peripheral lymphoid organs, and here both, immature and mature T cells reside (1, 2). CMJ is the site of progenitor immigration and the mature single-positive (SP) thymocytes emigration. The CMJ is also a place where the committed progenitors of medullary thymic epithelial cells (mTECs), termed junctional TECs, can be found (3, 4). Each subcompartment of the thymus contains several subtypes of TECs as well as dendritic cells (DCs), mesenchymal cells (MCs), and endothelial cells (ECs) (4–12). Additionally, B cells, natural killer (NK) cells, fibroblasts (Fbs), and macrophages (MFs) are present in the thymus (4, 8, 11, 12). These cells collectively establish and maintain the thymic microenvironment, which supports the differentiation of T cells (4, 6, 7) (**Figure 1**).

In the medulla, mTECs and medullary fibroblasts (mFbs) form a reticular structure where SP thymocytes are located and where they develop tolerance to self-antigens presented mTECs and mFbs (4). The presence of DCs and B cells in the medulla also contributes to the induction of T cell tolerance (4, 11, 13, 14). Part of the thymic ECs is encircled by pericytes, specialized fibroblast-like cells that express actin and contractile like the smooth muscle cells (4). Besides stromal cells, a range of uncharacteristic cells structurally similar to the epidermal or ciliated epithelium, neuroendocrine, muscle, or nerve cells is also present in the thymus (4). These cells can represent the subpopulations of differentiated mTECs forming Hassall’s corpuscles, neuroendocrine cell-like mTECs, and thymic tuft cells (4, 7). It is assumed that such a high diversity among mature mTECs might be the basis for their contribution to producing an assorted collection of self-antigens for the self-tolerance formation (7).

TECs are embedded in a 3D mesh structure. Together with MCs, TECs produce the thymic extracellular matrix (TECM), primarily composed of collagen type I and IV, fibronectin, and laminin (15, 16). TECM acts as a reservoir for soluble factors, that are essential for maintaining vital molecular pathways important for thymus organogenesis and T cell development (6, 16, 17). The thymus also hosts some tissue-specific progenitor/stem cell populations, especially thymic epithelial progenitor/stem cells (TEPCs/TESCs) (7, 18–27), mesenchymal stem cells (MSCs) (28–33), and lymphoid progenitor cells (LPCs) (10, 12, 34–36). At least part of LPCs probably is stem cells and resident radioresistant intrathymic stem cells (RTSCs) (37–45, 47–71).

The early studies identified TEC progenitors in murine embryonic thymic primordia and provided evidence that mTECs and cTECs share a common origin. These TEC progenitors might generate all known TEC subtypes *in vivo* and were sufficient to fully reconstitute the thymic epithelial microenvironment that supported normal T cell development (18–20). Later studies have reported that embryonic TEPCs expressing cortical markers can generate both cTECs and mTECs (21–23). Several groups have also reported the identification of bipotent TEPCs in adult mouse thymus (24–27). However, it remains unclear if the populations of fetal and adult TEC progenitors are the same, and that should be additionally studied.

Throughout the differentiation and maturation of T lymphocytes, which constitute over 95% of the thymus, there are three critical activities with a significant influence on the development of each T cell bearing a unique T cell receptor (TCR): 1) the TCR α and TCR β gene rearrangement and expression; 2) the positive selection of T cells that can distinguish self-major histocompatibility complex (MHC); 3) negative selection eliminating T cells that are potentially autoreactive (13, 14). T cells that withstand the negative selection and recognize self-MHC finally become mature CD4^+^ or CD8^+^ SP non-autoreactive T lymphocytes and migrate to the periphery (14). As well as T cell maturation and differentiation are supported and directed by numerous cytokines forming an intrathymic cytokine network, their traffic inside the thymus is orchestrated by chemokines, chemokine receptors, and G protein-coupled receptors (GPCR) (6). Both chemokine and cytokine networks are maintained by stromal cells and TECs, including cortical and medullary TECs (6–8).

TECs provide most of the specialized thymic functions mediating different phases of T cell development. cTECs are essential for the thymocyte progenitors’ commitment to T cells by providing the delta-like ligand Dll4 for Notch receptors (43–45), constitutively expressed by TLPs (43, 47, 48). Further, they drive the thymocyte expansion at several stages of development by providing different growth factors and critical cytokines, such as interleukin 7 (IL-7) (48–50) and stem cell factor (SCF), among others (6–8, 37, 51). cTECs also regulate the positive selection of T lymphocytes by delivering a unique set of peptides produced by β5t, a thymus-specific proteasome subunit (52). mTECs, on the other hand, expressing chemokines CCL19 and CCL21, promote the migration of positively selected thymocytes from the cortex to the medulla, where they regulate their negative selection and development of Foxp3^+^ natural T regulatory cells (Foxp3^+^ Treg), invariant γδ T cells, and invariant NKT cells (13, 14, 53, 54). mTECs also regulate the accumulation of DCs, one of the critical hematopoietic components of the thymic microenvironment, and their positioning in the medulla by secreting the XCL1 chemokine (55–57).

Therefore, developing a functional, self-tolerant T cell repertoire involves the communication between developing thymocytes and cTECs, mTECs, and other stromal and hematopoietic thymus components, many subtypes of which were recently additionally identified both in mice and human using modern single-cell RNA-sequencing (scRNA-seq) analysis (58–63). The role of these newly identified thymic cell subtypes in thymus function and development should be clarified to understand how this new knowledge may contribute to the *in vitro* thymus bioengineering reconstruction and *in vivo* thymus regenerative strategies. The review, in particular, discusses these issues.

## Thymic Epithelial Cells

The thymic function mainly depends on the TEC compartment of the stroma. While cTECs control T cell commitment and their positive selection, mTECs provide mechanisms to form the central tolerance of these T cells. The crucial role of mTECs in the T cell tolerance forming depends on several factors: the primary autoimmune regulator (Aire) regulating the expression of several tissue-restricted genes, and the Aire-independent mechanisms regulated, in particular, by Fezf2 (7, 51, 64, 65). The thymic epithelial component during embryogenesis and in the postnatal thymus are also maintained by TEPC/TESC-mediated cross-regulatory signaling between Notch and Foxn1 (66–70).

Numerous investigations indicate that cTECs and mTECs have a common epithelial progenitor during fetal (18–22, 70, 71) and postnatal development (24–27). In the mouse fetal thymus, this precursor appears in the thymic primordium as early as generated from the third pharyngeal pouches (3PPs) (18, 21, 70, 71). Early experiments showed that in mice both cTECs and mTECs are generated from fetal TEPCs expressing surface determinants that are recognized by the mAbs MTS20 and MTS24 (18, 19), later identified as the Plet1 (placenta-expressed transcript-1) antigen (72). Three complementary studies have almost simultaneously reported that embryonic TEPCs expressing cortical markers CD205 and β5t, and expressing IL-7, can generate both cTECs and mTECs (21–23). Later, another study showed that embryonic CCRL1^+^ cTECs also contain cells with mTEC potential (73). These studies led to the conceptual model that TEC progenitors exist within the cTEC niche prior to committing to the mTEC lineage – serial progression model (74). Thus, both TEC subsets arise from TEPCs/TESCs that express markers associated with mature cTECs, particularly CD205 and β5t (21, 23, 71). This fact suggests that fetal TEPCs are associated with the development of cTEC lineage but that for mTEC lineage specification, additional signals are essential (71). Since there is the shared expression of surface antigens between cTECs and the bipotent TEPCs, identifying the cTEC-restricted sublineage of TEPCs is still unsolved (7, 71). Furthermore, despite the further characterization of the bipotent TEPCs in the adult thymus (24–27), the phenotype of these cells yet remains to be specified.

Mouse TECs express the surface protein Plet1 that marks TEPCs/TESCs located in the thymic parenchyma at the CMJ (72). In the mature thymus, these TEPCs/TESCs additionally express Ly51 surface protein and also can generate both cTECs and mTECs (26). Moreover, Plet1^+^ TEPCs/TESCs express CD326 (EpCAM) surface protein (26). Thus, in the adult mice, bipotent TEC progenitors are CD326^+^UEA1^−^Ly-51^+^Plet1+MHC class II^hi^, which comprises <0.5% of adult TECs (26). Human TECs do not express Plet1 but express CD326, and therefore, in combination with Foxn1, this marker was used for isolation of the human TEPCs/TESCs from the neonatal and postnatal thymus (75, 76).

**Figure 2:**
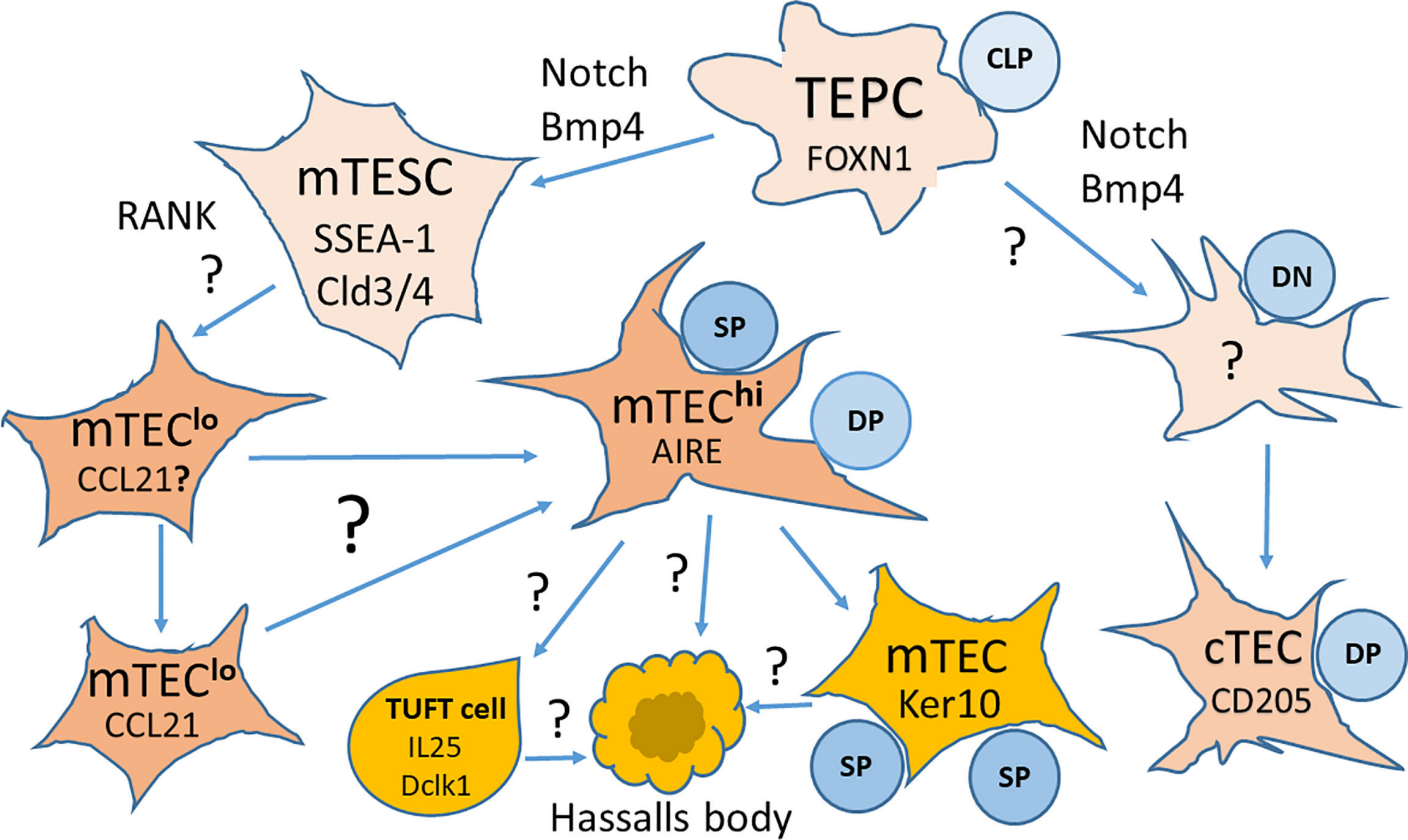
Key markers and pathways in the development of thymic epithelial cells (TECs) from bipotent thymic epithelial progenitor cells (TEPCs). TEPCs differentiate into medullary and cortical thymic epithelial cell lineages (mTECs and cTECs, respectively) that are regulated by Foxn1 expression. mTEC development goes through an intermediate stem cell stage (mTESCs), expressing the stem cell marker SSEA-1, and requires Notch signaling for the formation of mature Aire expressing mTECs^hi^. Other pathways to the differentiation of mTEC subsets are still a matter of intensive research. It is also yet not known whether cTEC development goes through a similar intermediate stage. CLP, Committed Lymphocyte Precursor; DN, Double Negative Thymocytes; DP, Double Positive Thymocytes; SP, Single Positive Thymocytes. Modified from Alawam et al., 2020 (71); the article is distributed under the terms of the Creative Commons Attribution License (CC BY).

Under the nonadhesive conditions, TEPCs in the mouse thymic cultures can form spheroid colonies, typical for cells with stem cell properties. These spheroid colonies (termed thymospheres) were EpCam^−^ and Foxn1^−^, and they generated both cTECs and mTECs (24). However, another group has reported that such thymospheres are formed by Foxn1^−^ EpCam^−^ MCs, and they have the potential to generate only adipocytes, but not TECs (31). Moreover, this study has shown that cells forming the thymospheres derive from the neural crest, and these structures can include bipotent TEPCs (31). These two studies were fulfilled with the mouse thymus, and there is still missing data concerning thymospheres of the human thymus, and therefore, they require further careful analyses. Since the existence of different TEPCs with self-renewing properties of stem cells remains discussed, additional studies are necessary to identify the earliest stages of TEC development in the embryonic and postnatal thymus that generate cTEC and mTEC lineages (71). cTECs and bipotent TEPCs share the expression of CD205, Ly51, and β5t. This sharing makes cTECs and TEPCs challenging to distinguish. However, recent data suggest that bipotent precursors have characteristics usually linked to cTECs before acquiring mTEC features (71). Key markers and pathways in TEC development from bipotent mouse TEPCs are presented in **Figure 2**.

The appearance of the earliest mTEC progenitors needs active Notch signaling in TEPCs, while further mTECs development is Notch independent, and continuing Notch activity supports the undifferentiated state of TEPCs (70). Moreover, Notch acts before NF-κB signaling to regulate mTEC lineage progression (70). These data suggest the complex influence of Notch signaling on TECs development and function. However, the mechanisms of Notch signaling on TEC development have not yet been clarified. Thus, Notch is a potent controller of the TEPCs and mTECs performance during T cell development. Therefore Notch is highly relevant for strategies generation/regeneration of the functional thymic tissue both *in vitro* and *in vivo*.

A minor population of TECs, expressing claudin 3 and 4 (Cld3/4) and SSEA-1 (stem cell marker) have been termed mTEC stem cells due to their self-renewal capabilities and the ability to differentiate into mTECs but not cTECs (7, 77). mTEC stem cells are also characterized a low expression of β5t and CD205 and a high expression of RANK (receptor activator of NF-κB) and lymphotoxin β receptor (LTβR) (7, 71). The generation of mTEC stem cells is Foxn1/Relb-independent as mTEC precursors emerge in Relb-deficient mice (78). However, their expansion and differentiation partially depend on LTβR, RANK, CD40, and p52 signaling (79–85). As shown, LTβR and RANK receptors are involved in the NF-κB pathway activation and control the proliferation and maturation of mTECs through an Aire-dependent way and the crosstalk with positively selected self-reactive CD4^+^ thymocytes (80, 81, 86–88). At this, lymphotoxin signaling is necessary for the expression of *Aire* and its downstream target genes. The failure of *Aire* induction in the thymus of lymphotoxin-deficient and LTβR-deficient mice contributes to autoimmunity against self-antigens normally protected by Aire (82). While only RANK signaling is essential for mTEC development during embryogenesis, cooperation between CD40 and RANK signals is required in postnatal mice (83). The RANK ligand (RANKL) produced by positively selected thymocytes is responsible for fostering thymic medulla formation, regulating the cellularity of mTECs by interacting with RANK and osteoprotegerin (84). Further expansion of the mature mTEC population requires autoantigen-specific interactions between positively selected CD4^+^ thymocytes bearing autoreactive T cell receptor (TCR) and mTECs bearing cognate self-peptide - MHC class II complexes. This interaction also engages the CD40 on mTECs by CD40L induced on the positively selected self-reactive CD4^+^ thymocytes (85). This antigen-specific TCR-MHC class II-mediated crosstalk between CD4^+^ thymocytes and mTECs is pivotal for generating a mature mTEC population competent for ensuring the central T cell tolerance (88). Recent RNA-seq analysis of transgenic mouse models has shown that self-reactive CD4^+^ thymocytes induce critical transcriptional regulators in mTEC^lo^ and control the composition of mTEC^lo^ subsets, including Aire^+^ mTEC^hi^ precursors, post-Aire and tuft-like mTECs (88). This interaction also upregulates the expression of tissue-restricted self-antigens, cytokines, chemokines, and adhesion molecules important for T cell development, and these interactions between self-reactive CD4^+^ thymocytes and mTECs are critically essential to prevent multiorgan autoimmunity (88). In addition, histone deacetylase 3 (HDAC3) is an essential regulator of mTECs differentiation (89), as well as STAT3 signaling is vital for mTECs expansion and maintenance (90, 91).

A fresh look at TEC heterogeneity, especially mTECs, provides the scRNA-seq technology analyzing the transcriptome patterns in combination with conventional surface marker analysis. Though both of these approaches have shown the identity of the main TEC populations, many unknown TEC subtypes, in particular, thymus tuft cells, were identified by scRNA-seq in both mice (62, 92) and humans (63). In the recent study authors additionally identified a population of Bpifa1^+^ Plet1^+^ mTECs that was preserved during thymus organogenesis in mice, and these mTECs highly expressed tissue-resident adult stem cell markers (93). Depending on the levels of MHCII and CD80, mTECs are broadly subdivided into mTECs^lo^ (*Cldn4*, lower levels of HLA class II) and mTECs^hi^ (*Spib, Aire, Fezf2*, higher levels of HLA class II). The mTEC^lo^ population includes the majority of mTECs. This population contains several subpopulations, including mTECs expressing a high level of the CCL21 chemokine and *Ccl21a* and *Krt5* genes (CCL21^+^ mTECs called also mTECs I) and stages representing Aire^+^ mTECs^hi^ (mTECs II) expressing the *Aire* and *Fezf2* genes (88). Recent data received with the help of scRNA-seq combined with lineage tracing and recovery from ablation have identified inside the mTEC^lo^ the short-living transit-amplifying cell population (TAC-TECs) that is the immediate precursor of Aire-expressing mTECs (94). These data also suggest that the TAC-TECs may also be the precursor of the *Ccl21a*-high mTEC population (94). However, yet it is unclear that Aire^+^ and Fezf2^+^ mTECs are developmentally related to CCL21^+^ mTECs. Aire^+^ mTECs^hi^ population further differentiates into post-Aire mTECs (mTECs III) expressing *Pigr* and *Cldn3* genes and thymic tuft cells (mTECs IV) producing IL-25 (62) and expressing *Avil* and *Pou2f3* genes (88). Post-Aire mTECs also contain Hassall’s bodies that contribute to the forming of thymic T cell tolerance as proposed (95). Finally, post-Aire mTECs become enriched for proteins classically associated with end-stage keratinocytes, such as involucrin (Ivl), Lekti, and a variety of different keratins, obtaining a corneocyte-like phenotype (7, 62, 71, 96–98). scRNA-seq analysis of the human thymus confirmed these four main mTEC subpopulations but added mTEC-myo and mTEC-neuro as two additional subpopulations presented in humans but absent in mice (63).

The essential feature that may be suitable to discriminate populations within mTECs^hi^ is the expression of the *Aire* gene, which is essential for the efficient deletion of self-reactive T cells (71, 86). Further, mTECs express the transcription factor Fezf2, which is required to activate some Aire-independent genes (64, 65). A high co-expression level of both factors, Aire and Fezf2, has been demonstrated by the cells expressing molecules associated with antigen presentation (65, 99). The co-expression of Aire and Fezf2 is a feature of the mouse and human mTECs (64, 100). In addition to that, within the mTEC^lo^ population, there has also been detected the Fezf2 expression, but without Aire expression (71, 81).

Aire^+^ mTECs continue their development past the stages of Aire expression and become typically differentiated keratinocytes (96, 97). These cells form well-known structures within the thymic medulla called Hassall’s corpuscles that can be identified by simultaneous keratin 10 and involucrin expression (71, 95). During ontogeny, Aire^+^ mTECs develop first due to the RANKL provision by DP thymocytes (83–85, 88). These Aire^+^ cells can progress to Aire^−^ cells that are characterized by lower levels of MHCII expression (7, 71, 96). However, post-Aire mTECs differ from other populations of mTECs MHCII^lo^ by the absence of CCL21 expression (7, 88, 97). A single-cell RNA sequencing analysis suggests the existence of two central populations of post-Aire mTECs presented by the keratin-10^+^ involucrin^+^ mTECs and the thymic tuft cells similar to the tuft cells originally described at mucosal sites (7, 62, 71, 101). Both types of tuft cells express *IL25*, *Trmp5*, *Dclk1*, and *IL17RB* genes (62, 101). The thymic tuft cell functions are still poorly understood. Since thymic tuft cells, unlike intestinal tuft cells, express high levels of MHCII (62, 101), it is possible that they have an active role in antigen presentation and thymic T cell selection (71, 92). Further, they might also regulate the innate immune networks, both thymic ILC and iNKT cells, within the thymus (17, 62, 102). Understanding how tuft cells and iNKT cells are connected to the intrathymic development of Tregs requires further studies. Although there is some evidence of DCLK1^+^ tuft cell presence within the human thymus (62), it is not clear whether human and mouse thymic tuft cells express a similar array of receptors and secreted factors.

The described heterogeneity of mTECs can explain how the thymus medulla supports the development of different T cell lineages, including conventional αβT cells, Foxp3^+^ Treg, invariant γδ T cells, and CD1d^−^-restricted iNKT cells (13, 53, 58–61, 71, 103).

cTECs are functionally very heterogeneous and among them are the thymic nurse cells (TNCs) represented by large epithelial cell complexes in which single cTECs enclose viable thymocytes. This unique feature of cTECs has been described in mice, where about 10–15% cTECs form such complexes, including four to eight DP thymocytes (104). cTECs that form TNCs have increased CD205, CXCL12, TGFβ, TSSP, and VCAM-1 compared to the cTECs that are not part of TNC structures (71, 105, 106). Analysis of DP thymocytes within the TNCs shows that they are enriched for cells that have undergone secondary TCRα rearrangements, indicating that they may provide an environment that enables efficient positive selection (71, 105).

Many studies demonstrated that the transcription factor Foxn1, a master regulator of TECs specification during the early stage of thymus development, is involved in the mechanisms of thymic involution. The high levels of Foxn1 expression are required for TEC development and maturation; moreover, thymopoiesis is dependent on the stable Foxn1 expression, and the reduced levels of Foxn1 have been observed in the aged thymus (2, 67–69, 107, 108). In the postnatal thymus, Foxn1 levels progressively and age-related decrease leading finally to the collapse of the thymic microenvironment and the complete failure of T cell production (69). However, the thymic involution can be reversed by an increased re-expression of Foxn1 (109). This thymic renewal is in line with restoring the thymic epithelial composition, effective thymopoiesis, a decrease of naïve T cells number in the periphery, and expansion of the memory T cells (15, 46, 109, 110).

In addition to Foxn1, several molecules and signaling pathways essential for T cell development were identified in the postnatal thymus in several transcriptome studies (69, 110). In particular, Wnt4 was described as a possible expression controller of Foxn1 in the early stages of thymic development (110, 111). Its expression is decreased with age matching the downregulation of Foxn1 (2, 108, 110, 111). Bone morphogenic protein-4 (Bmp4) is produced by thymic Fbs and ECs and participates in the early morphogenesis of the thymus (112). Receptors for Bmp4, BMPR I and II, are expressed in the postnatal thymus mainly by TEPCs. Bmp4 signaling mediates transforming growth factor-beta (TGF-β) through activation of *Smad4* (113, 114). The disbalance of TGF-β signaling molecules can reduce the capacity of TECs to support T cell development and, in this context, contributes to thymic involution (1, 17, 107, 112). Thus, understanding the molecular mechanisms regulating the levels of Foxn1 and other essential transcriptional factors provides critical knowledge for the development of TEC restoring strategies in thymus-compromised patients. Further studies in this promising area of research are essential.

## Thymic Mesenchymal Cells and Fibroblasts

MCs are the leading producers of the thymic extracellular matrix. This matrix ensures a structural mesh microenvironment for T cell migration and provides the main reservoir of cytokines and growth factors essential for epithelial and lymphoid progenitors during their differentiation and maturation. MCs have been described in all tissues and organs, where they have various mechanical and metabolic functions (37). Nevertheless, bone marrow MCs are the most explored since they are part of the hematopoietic stem cells (HSCs) niche, where the mesenchymal stem cells (MSCs) reside (113, 114). It has been shown that they can coordinate tissue regeneration and regulate the immune response (115–118).

On the other hand, it is little known about thymic MCs (TMCs) and especially their stem/progenitor cells (TMSCs) in the thymus function and development. Previous studies of the thymic stroma in both mice and humans using flow cytometry and bulk RNA-seq technology identified only several phenotypically distinct TMC subtypes (28–30). Modern research using gene expression profiles at single-cell resolution has shown a high heterogeneity among TMCs. In particular, this approach allowed identifying the unique transcriptional fingerprints of 12 non-epithelial stromal subtypes, including endothelial cells, vascular mural cells, neural crest-derived cells, mesothelial cells, and fibroblasts. Moreover, among the fibroblast population, at least 11 distinct capsular and medullary subtypes were identified, including capFb1a, capFb2b, and mFb1a as fibroblast subtypes with precursor potential and capFb3 to originate from mesothelial cells (63, 119).

TMCs contribute to the regulation of the TEC proliferation through the production of a set of cytokines, such as fibroblast growth factor (FGF) -7 and -10, insulin-like growth factor (IGF)-1, and -2, and retinoic acid (4, 28, 29, 115). Endosialin (CD248) positive TMCs play an essential role in revascularization during regeneration of the postnatal thymus after damage caused by infection (116). In contrast, fibroblast-specific protein 1 (FSP1) positive TMCs are required to preserve the mTEC compartment (4, 118, 119). The function of thymic MCs is similar to the function of other organ-specific MCs and includes the clearance of apoptotic cells in the thymus (29). Mouse TMCs that are negative for Foxn1 and EpCAM when cultured in nonadhesive conditions *in vitro* can form thymospheres, having adipocyte forming potential (31). TMCs can support the viability and differentiation of autologous thymocytes through direct contact, as has been shown in mouse co-cultures (117). On the other hand, there are indications of immunomodulating properties of human TMCs since they can reduce the proliferation of already activated thymocytes by 50%, as well as they can induce only a negligible proliferation of responding cells when tested in allogeneic co-cultures (29).

In the neonatal human thymus, some MCs are present that can be defined as trilineage stem cells, which include the previously postulated properties: i) attachment to the plastic surface, ii) expression of MSC-like surface markers, and iii) differentiation potential into osteogenic, chondrogenic, and adipogenic mesenchymal cell lineages in culture conditions. Some studies have also shown that neonatal TMSCs have immunomodulatory features and can differentiate into a cardiomyogenic lineage (29, 33). Moreover, neonatal TMSCs can express and *in vitro* secrete even more Sonic hedgehog (Shh), a proangiogenic and cardiac regenerative morphogen, than the bone-derived MSCs. Furthermore, in neonatal MSC organoid cultures, the expression of Shh ensures a cytoprotective effect for cardiomyocytes (33). TMSCs are negative for the hematopoietic surface antigens such as CD45, HLA-DR, HLA-ABC, CD34, CD38, CD40, CD40L, CD66, CD80, CD86, CD106 and positive for CD13, CD29, CD44, CD73, CD90, CD105, CD166 (4, 29, 37).

TMCs diversify at the early stage towards prethymic and intrathymic populations. The perithymic MCs form the thymic capsule, while the intrathymic populations differentiate into mFbs and pericytes (4, 118). However, how the generation of TMC diversity is regulated at the molecular level so far remains unknown (4). Neural crest-derived MCs in the adult thymus are presented by Fbs that are mainly located in the thymic capsule and medulla (4, 120). Thymic Fbs are important thymic stromal cells because of their large number and specific structure. They produce a collection of structural proteins such as collagens, as well as functional proteins that include FSP1, platelet-derived growth factor receptors α and β (PDGFRα and PDGFRβ), podoplanin/gp38, CD34, and epitopes for monoclonal antibodies known as MTS-15 and ERTR7 (4, 28, 30, 115, 118–121).

Capsular Fbs (capFbs) specifically express the surface protease dipeptidyl peptidase-4 (DPP4) or CD26, which is encoded by the differentially expressed gene *Dpp4* (4). This finding allowed the separation of the thymic fibroblast population on capFbs (DPP4^+^ gp38^+^) and mFbs (DPP4^−^ gp38^+^) (4, 122). Besides *Dpp4* expression, capFbs differ from mFbs by expression of *Pi16*, *Sema3c*, *Sema3d*, and *Aldh1a2* genes (4). Both capFbs and mFbs, are characterized by high expression of a set of fibroblast-associated genes, in particular, *Col1a1*, *Col3a1*, *Col6a1*, *Dcn*, *Lum*, *Mgp*, *Sparc* encoding the extracellular matrix proteins, *Serping1*, and *Serpinh1* encoding protease inhibitors, and *Htra1*, *Htra3*, *Mmp2*, *Mmp3*, *Mmp14*, *Mmp23* encoding extracellular proteases (4). Further, capFbs, in contrast to mFbs and other thymic stromal cells, have a higher level of Wnt family ligands and regulators (Wnt2, Wnt5a, Wnt5b, Wnt9a, Wnt10b, Wnt11, and Sfrp2 and Sfrp4) (4), suggesting that capFbs regulate cTEC development through the Wnt signaling.

mFbs are similar to fibroblastic reticular cells (FRCs) in the peripheral lymphoid organs, and these cells were previously described as thymic FRCs. However, they are a thymus-specific fibroblast subpopulation that is functionally distinct from FRCs of the secondary lymphoid organs (4). In adventitial layers surrounding the ECs and pericytes, the mFb subset of CD34^+^ podoplanin^+^ cells, better known as adventitial cells, has been identified (123, 124). mFbs predominantly express a set of genes that includes collagens (*Col6a5*, *Col6a6*), matrix metalloprotease-9 (*Mmp9*), metabolic enzymes (*Hmgcs2*, *Ltc4s*, and *Qprt*), and TGFb-binding proteins (*Ltbp1* and *Ltbp2*) (122). The lymphotoxin signal involving LTbR in mFbs regulates the adhesion molecules ICAM-1 and VCAM-1 (121, 124), suggesting a specific role of mFbs in the control of immune cell trafficking in the thymus (4). On the other hand, lymphotoxin, which in the thymus is produced by SP thymocytes, is required to develop mature mFbs and control the cellularity of Aire^+^ mTECs (4, 80, 83). Moreover, lymphotoxin promotes the differentiation of CCL21^+^ mTECs, Hassall’s corpuscles, and thymic tuft cells (4, 53). These facts illustrate the thymic cell crosstalk between lymphoid and stromal-epithelial cells providing the medullary microenvironment that controls the negative selection of SP thymocytes (4, 125). This intrathymic interaction is regulated by RANKL, the main mediator of the intrathymic crosstalk (83). RANKL is a TNF superfamily ligand and is expressed predominantly by mTECs and SP thymocytes (4, 83). Through signaling mediated by IkB kinase (IKK), NIK, and TRAF6, it activates the transcription factor NF-kB and thus induces Aire expression in mTECs and their further differentiation (4, 126). SP thymocytes also produce CD40L, which in cooperation with RANKL, promotes the development of mTECs expressing Aire (83).

Thereby, the use of gene expression profiles at single-cell resolution in contrast to conventional flow cytometry has demonstrated a high heterogeneity among thymic stromal cells. Single-cell transcriptome analyses showed dynamic changes in the frequency of these cells across an extensive range of developmental stages. However, the observed transcriptomic diversity of stromal subtypes is not fully supported by conventional flow cytometry due to a limited number of suitable cell surface markers. This limitation has yet hindered a comprehensive understanding functional role of nonepithelial stromal cell subtypes in the control of discrete stages of intrathymic T cell development. However, now there is no doubt that MCs and Fbs are the essential components of the thymic microenvironment, which is critical for its correct development and functioning.

## Thymic Lymphoid Stem Cells and T Cell Development

Early lymphoid precursor cells entering the thymus, the thymic lymphoid stem cells (TLSCs), are bone marrow migrants that in mice express low levels of CD4. In humans, they express CD34 and are negative for CD4, CD8, and TCRα/β (35, 37). These TLSCs can produce all known lymphoid lineages, including T cells, NK cells, B cells, and DCs (10, 34–37, 127, 128). In the bone marrow, the proliferation and fate HSCs are regulated by the stem cell factor (SCF) and its c-kit receptor in cooperation with Notch ligands and morphogenic factors, such as Wnt, Hedgehog, TGFβ, and BMP (34, 37, 127–130). They regulate the stem cell self-renewal and differentiation into multiple lineages (35, 127). The early TLSCs, which are bone marrow migrants, enter the thymus through the blood vessels in the CMJ area. They migrate consequentially to the thymic subcapsular zone (37), the thymic cortical, and medullary zones while differentiating first into immature DP (CD4^+^ CD8^+^) thymocytes. Finally, they become naive SP CD4^+^ and CD8^+^ T cells (**Figure 1**). Following this path, they undergo the positive and negative selection in the cortical and medullar regions of the thymus, respectively (2, 14, 37, 71). These intrathymic events are controlled by the direct interaction of thymocytes with the stromal-epithelial compartment. The chemokine, hormonal, and cytokine signals ensure the necessary conditions for the correct maturation, differentiation, and T cell trafficking through the thymus (4, 6, 8, 17, 34, 55, 56, 71).

In more detail, the development of thymocytes starts from early CD25^–^CD44^+^ TLPCs/TLSCs deriving from HSCs of the fetal liver or the adult bone marrow. The early stage of T cell maturation mainly occurs in the cortex of the thymus and is directed by contact with cTECs. A high level of chemokines, such as CCL25, CXCR4, CXCL12, Notch ligand DLL4, and cytokines IL-7 and SCF provided by these cTECs, is required for the development of these early thymocytes (2, 6, 15, 34, 37, 71). The CCL25 and CXCL12 chemokines ensure the growth and survival of TLPs in the cortex, the Notch ligand DLL4 enables the differentiation of TLPs into T cells (46–48, 131, 132), and IL-7 and c-kit ensure the proliferation of the immature T cells (47, 132, 133). Early TLPs go through the double negative 2 (DN2) stage when they express CD25 and CD44, proliferate, and downregulate CD44 to develop into the DN3 stage of CD25^+^CD44^–^ thymocytes. At this stage, they lose the B cell potential (15, 17, 128) and move through the cortex into the subcapsular area, and TCRβ undergoes rearrangement. DN3 cells further differentiate into DN4 CD25^–^CD44^–^ thymocytes. They actively proliferate and continue to develop into the DP CD4^+^CD8^+^ stage. DP thymocytes further rearrange the TCRα and finally express the mature TCRαβ complex. Thus, still immature T cells co-express CD4, CD8, and the TCRαβ complex in combination with CD3 (TCRαβ–CD3) (6, 15, 128).

In the outer cortex, cTECs activate the positive selection of the DP T cells with the help of MHC self-peptides and the TCR of maturing T cells (6, 15). This interaction of DP thymocytes with cTECs initiates the survival or cell death of DP thymocytes (6, 7, 15). Intrathymic location of CD4^−^CD8^−^ TLPs is regulated by chemokine CXCR4 and chemokine ligand CXCL12 interaction, while the maturation of these TLPs expressing pre-TCR requires the CXCR4–CXCL12 interaction together with Notch signaling to control β-selection (71, 131). In the later phase of thymocyte maturation, CXCL12 retains CD4^+^CD8^+^ thymocytes in the cortex to undergo correct maturational stages, including positive selection (69, 71, 131). cTECs can also support the positive selection of CD8^+^ T cells mediated by MHC class I. However, the processing and presentation of peptides associated with molecules of MHC-I require the expression and degradation of thymic proteasomes in cTECs and the presentation of proteasomal catalytic subunit β5t (7, 52, 71, 106).

After positive selection, DP thymocytes develop into SP CD4^+^CD8^–^ or CD4^–^CD8^+^ T cells and they bind MHC II or MHC I, respectively (7, 15, 71, 128). These cells are transferred to the medulla, and this transfer is regulated by chemokine ligands CCL21 and CCL19 on mTECs (7, 15, 71). In the medulla, T lymphocytes with the self-reactive TCR undergo negative selection mediated by mTECs expressing a set of tissue-restricted antigens regulated by Aire and Fezf2 (6, 7, 14, 15, 64, 65, 71). In addition, thymic DCs also partake in the T cell selection through the expression of endogenous antigens or the presentation of antigens from other cell types (11, 15, 29, 55, 56, 122). In the medulla, the self-reactive T cells are removed by negative selection, and conventional regulatory T cells (Tregs) and Foxp3^+^ Tregs that express diverse TCR repertoires are developed (15, 54, 123, 125). Finally, naïve CD4^+^ and CD8^+^ SP T cells and Tregs migrate from the medullar to the peripheral lymphoid tissue and circulation (15, 128).

In addition to the development of the conventional TCRαβ^+^ T cells, the thymus also supports the progress of innate-like TCRγδ^+^ T cells (γδ T cells) (54, 71). These cells do not require antigen-specific communications with the stromal microenvironment for development (71, 103). In mice, γδ T cells are generated at the transient DN2a-DN2b stages from the same DN1 early T cell progenitors (ETPs) (128), which appear as multipotent TLSCs (**Figure 1**). These γδ T cells are produced at some periods of ontogeny, and they are distributed to epithelial and mucosal tissues (15, 54, 103, 128). The development of γδ T cell populations was recently reviewed in detail by Parker and Ciofani (103). TLSCs can also contribute to the generation of the thymic B cells, DCs, NK cells, and MFs, at least in mice (10, 12, 128, 133), confirming their multipotent stem cell potential.

Human TLP development includes a CD4^+^ SP stage, after which they diverge to the αβ or γδ lineages (15, 58, 59, 61). Human CD4^+^ SP αβ thymocytes are found in the cortex, and they are turned to the DP form and then move to the medulla generating CD4^+^ and CD8^+^ SP T cells (2, 15, 103). Human negative and positive T cell selection also occurs in the cortex and medulla, probably similar to the selection in the mouse thymus (15, 58, 59). However, these events concerning the human thymus are yet under discussion. Another difference is that humans are born with an entire T cell repertoire, and T cell memory is formed during childhood (15, 58). The thymus suffers age-related involution throughout life, which is associated with an essential reduction in the proliferation and differentiation of early TLPs, altered T cell differentiation, and atrophy of the epithelial compartment (15). The malfunction or even impairment of thymus development is linked with several diseases like the DiGeorge Syndrome (DGS), Foxn1 deficiency, graft-versus-host disease (GVHD), HIV infection, or autoimmune diseases (1, 2, 9, 15).

## Thymic Innate Lymphoid Cells

Innate lymphoid cells (ILCs) are tissue-resident cells. They comprise NK cells and lymphoid tissue inducer (LTi) cells triggered through receptors for pathogens or inflammatory cytokines but not through the BCR or TCR antigen-specific receptors (128, 134–136). They are usually located at the mucosal barrier sites, and they regulate homeostasis and tissue repair in non-barrier organs (128, 137, 138).

ILCs are very heterogeneous, and they have been grouped into subsets according to their surface marker expression, cytokine profiles, and transcription factors, similarly to the T cell classification, on ILC1/NK, ILC2, and ILC3/LTi (128, 139, 140). ILC1 are conventional NK cells and helper ILC1. They are identified by the production of interferon γ (IFN-γ) in response to IL-12, IL-15, and IL-18. ILC2 generates cytokines IL-5, IL-9, and IL-13 in response to stimulation by alarmins, IL-25, IL-33, and TSLP, and they depend on the expression of GATA3. Finally, ILC3 is characterized by the secretion of IL-17 and IL-22 in response to stimulation by IL-23 and IL-1β (128). IL-22 is critically important for thymus recovery after damage, and since ILC3 cells are highly resistant to damage, they play an essential role in thymus organogenesis and regeneration (144, 145). ILC3 cells are regulated by the transcription factors RORγt and RORα (127, 140–142). This population also contains an LTi family. LTi is generated during embryogenesis and facilitates the development of secondary lymphoid tissues (128, 143).

In general, the ILC1 population is essential for the clearance of intracellular pathogens, ILC2 for helminth infection and allergen-induced chronic airway inflammation, and ILC3 for gut immunity and for establishing gut tolerance and mucus secretion (128). Concerning the thymus, ILC3 is dominant in the embryo, and ILC2 in the postnatal thymus (128). A detailed analysis of ILC development in the thymus is presented in the review by Shin and McNagny (128), and it is shown in **Figure 1**.

## Thymic Radioresistant and Radiosensitive Cells

As early as 1975, Kadish and Basch were the first to report that the thymus of adult mice contains cells resistant to radiation. These cells can temporarily restore the thymus cellularity after sublethal total body irradiation (38). This rare population of radioresistant thymic cells is CD4^−^CD8^−^ intrathymic TLPs (previously known as L3T4^−^Lyt2^−^), probably located in the subcapsular part of the thymic cortex (37–39). The significance and biological role of radioresistant intrathymic TLPs for the thymic function is yet unclear, and several research groups are tackling this intriguing research area (37–45, 47–71, 146–151).

The most important index of cell radioresistance is their stability to interphase death, which is measured by the dose irradiation that causes 63% of cell death (D_0_). The radioresistance is an essential peculiarity of the resting cells. The radioresistance of lymphocytes is in the range of 1-10 Sievert (Sv), and it varies with the maturation stage and the cell subpopulation type. Concerning the thymus, a small population of intrathymic TLPs represents the most radioresistant lymphoid cells playing a special role in the post-radiation restoration of the thymus (37–45, 47–71, 146, 147). Studies of Shichkin’s research group, which were recently revisited and updated (37), have shown that radioresistant intrathymic TLPs produce an autocrine thymocyte growth factor (THGF). The target cells for THGF are the radioresistant TLPs, which have D_0_ of more than 50 Sv (37). With a radiation dose of more than 15 Sv, these TLPs persisted in an inactive or low activity state for a long time. However, exogenic THGF or its combination with IL-2 can activate and increase their proliferation. The gamma-irradiation at the dose of 12 Sv induces the secretion of THGF by the radioresistant cells, and this cytokine then supports the self-regulating proliferation of these cells in autocrine manner (37, 41, 146, 152, 153). They are probably early intrathymic TLPs, persisting at the DN1/DN2 stage as resting resident tissue-specific stem cells (**Figure 1**), which are the direct target-cells for TGHF. This assumption is supported by the data showing the existence of the radioresistant subpopulation of TLPs at the DN2 stage of thymocyte development that proliferate after irradiation in an IL-7-dependent manner and generate the conventional thymocytes. Moreover, their differentiation recapitulates normal thymic ontogeny (148). These data provide evidence concerning the specificity of THGF-dependent proliferation of radioresistant TLPs and successive change of THGF-sensitive stage to THGF/IL-2-sensitive stage (37).

Furthermore, there is evidence that THGF-dependent cells are self-renewing intrathymic CD4^−^CD8^−^ stem cells, activated by THGF and damage factors such as irradiation. THGF is probably a member of the SCF superfamily (37). Therefore, we are bringing together but not identifying THGF with IL-7, SCF, and GM-CSF. On the other hand, some authors have shown that the intrathymic progenitors are multipotent and may generate not only a T cell lineage but also NK cells (154), DCs (155), MFs, and B cells (156). These cells could, in turn, secrete IL-7, SCF, and other cytokines and thereby support T cell development during the reconstitution of the irradiated thymus.

A recently defined subpopulation of radioresistant TECs (149) may also contribute to the post-radiation restoration of the thymic function by producing these cytokines and providing signaling pathways essential for intercommunication with radioresistant TLPs. In particular, chemotherapy and radiotherapy, cytotoxic therapies cause apoptotic death of radiosensitive thymocytes and TECs (71). Following sublethal irradiation of mice, both cTECs and mTECs are reduced, indicating the radiosensitivity of most TECs (71, 149). However, after irradiation, some TECs can produce chemokines such as CCL19, CCL21, and CCL25 that are important for the recruitment of TLPs (71, 157). Furthermore, ECs are also radioresistant and can recruit TLPs (71). Therefore, together with radioresistant TLP and some TECs, ECs are essential in post-damage thymic regeneration.

Recent studies have shown that ILC3 and Th17 cells produce a cytokine (IL-22) that is critical for the thymic epithelial compartment recovery after high-dose chemotherapy or irradiation damage (144, 145, 151). IL-22 increased the number of TECs through the Stat3-dependent signaling pathway in the mTEC1 murine TEC line (158). Defects of IL-22 production delay thymus recovery in irradiated mice and act on the expression of genes associated with thymic function, such as *Foxn1*, *Aire*, and *Kgf*. In contrast, the use of IL-22 facilitates the repair of TECs, increases the number of T cells, increases the Aire level, and increases the proportion of natural regulatory T cells in the thymus (158), suggesting the critical role of the IL-22/Stat3/Mcl-1 pathway in the regeneration of TEC compartment after the irradiation damage. Following the total body irradiation or targeted irradiation of the thymus at the critical depletion of DP thymocytes, the intrathymic IL-22 level has been increased, suggesting a link of IL-22 with mechanisms of endogenous recovery (144, 151) and that is very similar to the effect of THGF (37). Production of IL-22 following damage is attributed to radioresistant thymic LTi/ILC3 cells, which were present in increased numbers following thymic insult, and RANKL molecule was implicated in thymus regeneration, which expression was upregulated by radioresistant LTi/ILC3 (71, 144).

In addition to the IL22 and THGF potential, a recent study highlighted the involvement of Bmp4 in thymus recovery following damage (112). Bmp4 is produced by multiple stromal cells within the thymus, including Fbs and ECs. However, following the total body irradiation, Bmp4 expression was upregulated only by ECs, resulting in increased cTECs, and an increase in Foxn1 levels and its target genes such as *Dll4*, *Kitl*, and *Cxcl12* (71, 112). Therefore, in this way, ECs involve Bmp4 to initiate thymus recovery. Since the number of ECs remains unchanged in the thymus after total body irradiation, ECs appear to be radioresistant thymic cells similarly to ILC3/LTi and resident THGF-sensitive TLPs. These new data provide a fresh look at the role of different radioresistant thymic cell populations in the thymus post-radiation regeneration and thymic function recovery.

## Inthrathymic Cytokine Network

Thymocyte differentiation is regulated by direct contact with the stromal-epithelial microenvironment and responds to various cytokines produced by thymic stromal and lymphoid cells. IL-7, produced by TECs, is the key regulator of T cell maturation, differentiation, and survival in the early stages of their generation (37, 159). However, many other cytokines, usually presenting in the periphery, can also be found in the thymus. Since the thymus is a relatively closed organ for macromolecular migration into/from the organ, it is likely that the thymic cytokine network is adapted for the thymus itself and that cytokines do not leak to the periphery. However, for many intrathymic cytokines, their biological role is still unclear.

Various cytokines, such as IL-1, IL-3, IL-6, IL-7, IL-8, IL-12, IL-15, IL-25, as well as SCF, GM-CSF, G-CSF, M-CSF, TNFα, and TFRβ that are constitutively secreted by TECs, MCs, Fbs, and other stromal elements, have been identified in the thymus during the past decade. In addition, many cytokines, such as IL-1, IL-2, IL-3, IL-4, IL-5, IL-10, IL-17, IL-22, IL-23, THGF, IFN-γ, GM-CSF, G-CSF are the constitutive and/or inducible products of the thymic lymphoid populations, mainly DN TLPs, DP thymocytes, SP T cells, and thymic ILCs (6, 8, 16, 34, 35, 62, 108, 127, 132, 144, 146, 153, 159, 160).

TECs and T cells are two central populations of the thymic cells, which produce cytokines in the thymus. However, all thymic cells can secrete cytokines spontaneously or after stimulation. Among TECs, subcapsular and mTECs are more active cytokine producers than cTECs, and the cytokine profile of TECs is very close to the one of peripheral MFs and monocytes (2, 6, 7, 37, 71, 161). Although, when compared to the stromal thymic elements, thymocytes are relatively weak cytokine producers but being the most prominent population, their contribution to the intrathymic cytokine network is substantial. The cytokine-producing ability of thymocytes is gradually reduced during their maturation from the stage of DN CD44^+^CD3^−^CD4^−^CD8^−^ TLPs to the stage of immature DP CD3^lo^CD4^+^CD8^+^ cortical thymocytes, and it is completely blocked in the latter DP stage. However, the capacity of thymocytes to produce cytokines and respond to their action is restored at the CD4^+^ and CD8^+^ SP stages of thymopoiesis following the completion of the selection process (37, 161, 162). The factors that activate and regulate the intrathymic cytokine secretion are still a matter of discussion, and they require further evaluation. Intercellular contacts, especially between TECs and thymocytes, play an important activation and modulating role in cytokine production in the thymus. In the mature thymocytes, the cytokines are produced in response to TCR-CD3 receptor complex binding (160, 162).

Inside the thymus, cytokines act as short distance factors, and their biological effects are determined by the expression of cytokine receptors on thymic cells. Some thymic cytokines can act as paracrine, and others appear as autocrine factors. IL-7 and SCF are examples of paracrine thymic cytokines that are produced by TECs and thymic MCs and induce the growth and differentiation of CD4^−^CD8^−^ TLPs (37, 159, 163). INFγ is another example of the paracrine cytokine, which is produced by mature SP thymocytes and participates in the control of T cell maturation and differentiation (6, 37, 161). IL-2 and IL-4, for which the producers and targets are thymocytes at the different stages of maturing, can act both in a paracrine and autocrine manner. At the same time, THGF probably appears only as the autocrine factor for which the producers and targets are radioresistant intrathymic CD4^−^CD8^−^ TLPs (37, 41, 161). While SCF, IL-7, and THGF are essential to promote the proliferation and survival of CD4^−^CD8^−^ intrathymic TLPs, IL-2 and IL-4 are more specific for the final stages of T cell development (6, 37, 161). On the other hand, some CD4^−^CD8^−^ stages of intrathymic TLPs are also sensitive to IL-2 and IL-4, while IL-7, together with IL-12, IL-22, IL-23, and IFN-γ actively contribute to the negative selection and final stages of thymocyte differentiation (160).

SCF plays a key role in bone marrow hematopoiesis and lymphopoiesis (163); this growth factor is produced by thymic stromal cells, preferably by TECs and MCs, and SCF can directly stimulate the proliferation of CD4^−^CD8^−^ TLPs (71, 164, 165). Early TLPs exhibit high expression of the c-kit receptor for SCF. Therefore, the SCF/c-kit complex is essential during the early stages of thymopoiesis (35, 127, 128, 161), similar to IL-7 and THGF, suggesting that these cytokines belong to one functional group. Key signaling molecules of the intrathymic cellular network are summarized and presented in **Table 1**.

**Table 1:** Key signaling molecules of intrathymic cellular network.

Molecule	Cell expression	Functions in thymus	References
CD205	cTECs	Apoptopic cell clearance	(71)
β5t	cTECs, TEPCs	Thymic proteosome component, CD8^+^ T cell selection	(71)
PRSS16	cTECs	Thymus specific serine protease, CD4^+^ selection	(71)
DLL4	cTECs	Notch ligand, regulator of T cell commitment and β selection	(71)
CXCL12	cTECs	Chemokine ligand for CXCR4, regulation of β selection	(71)
CCL21	mTECs	Chemokine ligand for CCR7, regulator of cortex to medulla migration of SP thymocytes	(71)
CCL25	cTECs, mTECs	Chemokine ligand for CCR9, recruitment and positioning of TLPs, regulator of CD4^+^CD8^+^ thymocyte migration	(71)
LTβR	cTECs, mTECs	Ligand for lymphotoxin, regulator of mTEC and thymic endothelium development	(71)
Aire	mTECs	Tissue restricred antigen expression, tolerance	(71)
Fezf2	mTECs	Tissue restricred antigen expression, tolerance	(71)
RANK	mTECs, mTEPCs	mTEC development	(71)
Relb	mTECs	mTEPC development	(71)
IFNγ	Activated T cells, NK cells	T cell maturation and differentiation	(6)
SCF	cTECs	Maintenance of TLPs	(71)
THGF	Self-renewing TLPs	Activation and proliferation of self- renewing TLPs	(36, 152, 153)
IL-1	TECs, Macrophages	T cell activation and growth	(6)
IL-2	Activated T cells	T cell activation and development	(6)
IL-4	Activated T cells	T cell growth factor	(6)
IL-6	Macrophages, fibroblasts	T cell maturation and development	(6)
IL-7	cTECs and mTECs in adult thymus, TEPCs in embryonic thymus, stromal cells, DCs	Proliferation of TLPs	(6, 71)
IL-9	Activated T cells	T cell growth factor	(6)
IL-12	T cells	Maintenance of thymus integrity and function	(6)
IL-15	mTECs	Regulation of iNKT cells	(71)
IL-17	T cells	Activation of CD4^+^ T cells, production of Treg17 cells	(6)
IL-21	Activated CD4^+^ T cells	Differentiation of CD4^+^ T cells, development of Treg17 cells	(6)
IL-22	Th17 cells, γδ T cells, NKT cells, ILCs	Proliferation and survival of TECs, Thymus regeneration	(6, 142, 143)
IL-25	Thymic tuft cells	Regulation of intrathymic ILCs and iNKT cells	(71)
TGFβ	Activated T cells	Inhibition of IL-1-, IL-2- and IL-7-dependent proliferation of thymocytes	(6)
TNFα	Macrophages	Promotion of T cell proliferation	(6)
TSLP	TECs, DCs	Promotion of Th2 cell differentiation of CD4^+^ naïve T cells, activation of ILCs	(6)

DLL4, Delta like 4; LTβR, Lymphotoxin beta Receptor; Aire, Autoimmune Regulator; RANK, Receptor Activator of Nuclear Factor κB; ILCs, Innate Lymphoid Cells; iNKT, invariant Natural Killer T Cells; SP, Single Positive; DCs, Dendritic Cells; cTECs, cortical Thymic Epithelial Cells; mTECs; medullary Thymic Epithelial Cells; TEPCs, Thymic Epithelial Progenitor Cells; TLPs, Thymic Lymphocite Progenitors; Th, T helper; Treg, T regulator; IFNγ, Interferon gamma; SCF, Stem Cell Factor; THGF, Thymocyte Growth Factor; IL, Interleukin; TGFβ, Transforming Growth Factor beta; TNFα, Tumor Necrosis Factor alpha; TSLP, Thymic Stromal Lymphopoietin.

## Thymus Reconstitution Strategies

As early as 1961, Jacques Miller first reported the importance of the thymus for the development and function of the immune system function. Though many aspects are still a matter of intensive research, it is accepted that impaired thymus function can lead to various dramatic consequences, including the development of autoimmune diseases, increased susceptibility to infection, high risk of cancer, as well as a decreased immune response to vaccination (9, 166, 167). Complete thymectomy in neonates, especially if thymectomy was done at the age below 1 year, leads to the development of age-associated diseases, such as autoimmune and neurodegenerative diseases and atherosclerosis, and such patients have a persistent imbalance of naïve T cells in the periphery (9, 166, 167). During standard surgical procedures concerning congenital heart diseases, the thymus becomes biological waste. It can be used as a source of autologous tissue-specific stem cells for personalized treatment of thymectomized infants. With this, actual challenges are the optimization of thymectomy procedure in infants, collection and cryopreservation of thymic tissue, preparation of thymic stem cells, their clonal expansion, and development of robust protocols for autologous stem cell-based therapy (9, 37).

Current technologies for restoring the thymic function are focused mainly on using TEPCs/TESCs for remodeling functional thymic organoids *in vitro* or *in vivo* (15, 37, 71, 168, 169). The main obstacles to translating these technologies into medical practice are the small numbers of TESCs in the human thymus, difficulties of their isolation, purification, especially expansion *in vitro*, and formulation of the fully functional thymic organoids *ex vivo* (9, 37). The absence of effective methods for maintaining undifferentiated functional TESCs *in vitro* and the preferential growth of Fbs in such cultures, yet represent a significant challenge for the study and possible application of the cryopreserved TESCs (9, 37, 75, 76, 170).

**Figure 3:**
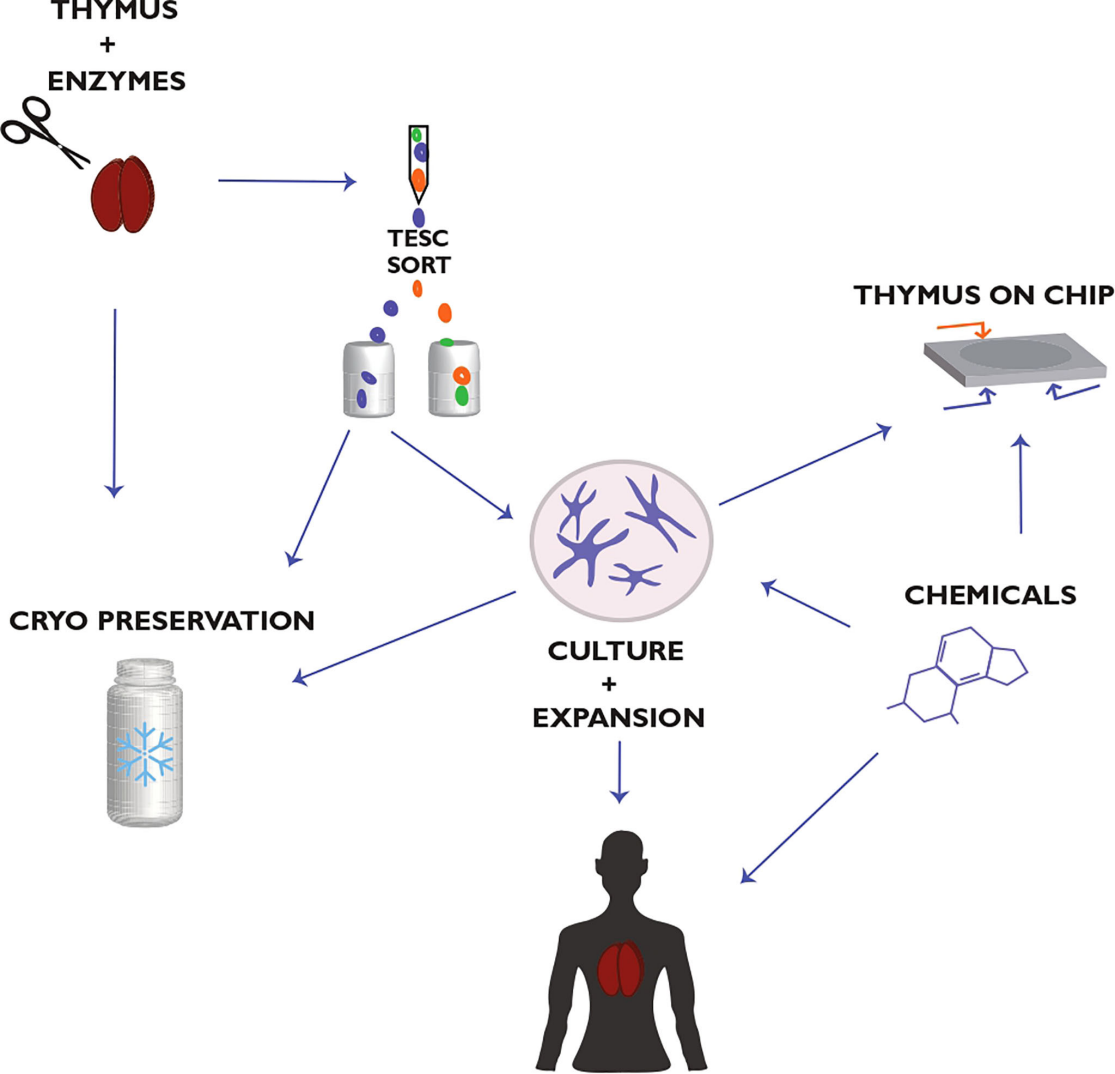
Thymic epithelial stem cell (TESC)-based strategy for thymus regeneration with small chemical compounds (SCC). This strategy proposes the collection, preparation, and cryopreservation of primary thymic tissue and TESC-enriched cell samples. These thymic samples are used further to select TESC-specific SCC that can regulate the differentiation and proliferation of human TESCs and support their clonal expansion. The selected SCC are tested also for supporting thymic tissue growth *in vitro* as well as for reconstitution of thymic function in terms of differentiation, maturation, and tolerance of autologous T cells. The use of microfluidic chips in combination with human 3D thymic organ cultures (Thymus-on-Chip devices) to assess SCC specificity and toxicity is essential to accelerate drug development for thymus-compromised patients. Actual challenges are optimizing the thymectomy procedure in patients to preserve a thymic fragment for consequent postsurgical thymus regeneration and the quality life monitoring of thymectomized patients concerning their resistance to infections, allergies, autoimmune, oncological, and other diseases associated with the impaired thymic function. Modified from Shichkin and Antica, 2020 (9); the article is licensed under a Creative Commons Attribution 4.0 International License.

Current approaches exploring how to reach a stable growth of TESCs *in vitro* apply the use of serum-free culture media, adding TESC-supporting compositions of growth factors, adding supplements that can inhibit the growth of other cell types, the use of nonadhesive materials to generate 3D TEC cultures (9, 15, 37, 71, 169). The use of small chemical compounds (SCC) blocking or enhancing signaling mediated by specific protein kinases and thus regulating the differentiation and clonal expansion of stem cells can be an additional practical component to reach this aim (9, 37, 171–173). Many studies have tested the target-specific SCC using human pluripotent ESCs, iPSCs, and HSCs (172). These studies validate the use of SCC for tissue engineering *in vitro* and for boosting the regenerative potential of stem cells *in vivo*. However, optimal SCC for TESCs has yet to be found and structurally optimized to achieve adequate efficiency and low toxicity (**Figure 3**).

Different molecules such as keratinocyte growth factor (KGF), Flt-3 Ligand (Flt3L), IL-7, IL-21, IL-22, RANKL, and growth hormones have been proposed as effective approaches to boost the endogenous thymic repair since these molecules are essential in thymopoiesis and for the maintenance of the epithelial compartment. In particular, the administration of IL-7, Flt3L, KGF, IL-21, and IL-22, have been used to support the thymus regeneration after high dose radio-chemotherapy injury (15, 144, 145, 151, 158). A clinical trial demonstrated that CD4^+^ and CD8^+^ T cells are increased by treating patients with IL-7, although an essential effect on thymic growth was not observed (15). A clinical trial with KGF to evaluate T cell recovery in HIV patients has not identified essential effects on thymic size or production of T cells in the thymus (15). In contrast, growth hormones have been influential in the reconstitution of the immune system and enhanced recovery of the thymus in HIV patients, demonstrating a higher thymic mass and numbers of circulating naïve T cells and CD4^+^ T cells (15).

The first well-described pathway for endogenous thymus regeneration after the damage was centered on the production of IL-22 by resistance to injury ILCs (144, 151). Additionally to IL-22, ILCs also increase the production of RANKL that, in an autocrine manner, regulate IL-22 secretion by ILCs after thymic damage (144, 151). A second pathway is the IL-22-independent and connected with the production of BMP4 by ECs, which, similar to ILCs, are highly resistant to damage (112). The high radioresistance of these cells allows them to respond to activation by yet unknown signals and produce BMP4, which stimulates TECs to induce Foxn1 expression that controls DLL4 and Kit ligand transcription. These factors are critical for thymopoiesis and can control the thymic size (112, 151). The use of cytokines together with SCC that are essential for thymic regeneration *in vivo* may provide much more benefits for different groups of patients with thymic involution, including aging people representing the most significant population needed in this medical treatment, than approaches using TESC-based technologies *in vitro* to generate the functional thymic organoids.

Several studies have evaluated the effectiveness of bone marrow-derived lymphoid progenitors, which were admixed with HSCs to accelerate and enhance immune rejuvenation. The limited supply of these lymphoid progenitors restricts this approach. However, with the development of new *in vitro* systems that use Notch-1 stimulation to generate T lineage-committed progenitors, 3D culture systems, and cell feeder-free culture conditions, this challenge can now be overcome (15, 45, 171–175).

In the last decade, essential efforts have been undertaken to generate *ex vivo* the functionally complete thymic microenvironment or thymic organoids that can be transplanted into patients. The advancement of these directions provides a promising framework for generating a *de novo* thymus from epithelial progenitors or pluripotent stem cells (15, 168, 169, 176). TEC-like cells, which are suitable for these modern technologies and can support T cell development, can be generated from Fbs with the help of targeted expression of *Foxn1* (174), demonstrating promising capabilities of these inducible TECs (iTECs).

Several studies have demonstrated the possibility of generating the functional thymus microenvironment from single mouse embryonal TESCs/TEPCs (19, 21, 175, 176) or iPSCs (174) injected under the kidney capsule in mice. These experiments illustrate the multipotency of the early TESC/TEPC and support ongoing efforts in the generation of the functional thymic organoids *in vitro* using epithelial progenitors and/or iPSCs. However, diversification of some thymic cell lineages essential for thymic function begins very early in the embryonal stage, and some of them have different embryonal precursors. Therefore, considering these factors, it appears problematic to develop a fully functional bioengineering thymus, which could provide the correct negative and positive selection of T cell repertoires only from epithelial precursors, and a combination at least with thymic mesenchymal and endothelial precursors may be needed. Moreover, the mouse thymus development and function are not entirely equivalent to the human thymus. Therefore, the knowledge received with mouse models may have a set of restrictions that should be considered when translating into medical practice. Considering these restrictions, combining different approaches instead of a single may better unlock the thymic regenerative potential and be more suitable for translating to the clinic. More detailed analyses of current thymic regenerative strategies are provided in the recent reviews (15, 177, 178).

## Conclusion

Thymus function is based on the fine-tuning of specialized stromal, mesenchymal, epithelial, and endothelial cells and their products, which are essential for the continuous output of immunocompetent T lymphocytes. Although thymus involution occurs at puberty and its function decreases with aging, there is a great potential for restoring its function by either cell transplantation, regenerative therapy *in vivo*, or bioengineering strategies. Identifying cells and molecular factors that are important for differentiation, positive and negative selection, and generation of naïve T cells and translating of the experiments from the mouse models to humans is necessary for restoring a damaged thymus. The clinical application of the stem cells from adult tissues, that best resembles the *in vivo* conditions, will allow a better immune response in patients after iatrogenic thymus damage, patients born with thymus deficiency, or aged persons, increasing the efficiency of immunotherapy, including vaccination procedures.

## Author Contributions

VS reviewed the literature, wrote and redacted the manuscript, and designed and redacted the illustrations. MA redacted the manuscript and designed and redacted figures. All authors contributed to the article and approved the submitted version.

## Funding

This work was supported by the Croatian Science Foundation Grant IP-2020-02-2431, The Terry Fox Foundation Zagreb Run and Croatian League against Cancer, and by the Scientific Centre of Excellence for Reproductive and Regenerative Medicine (Grant Agreement KK01.1.1.01.0008 that is funded by the European Union through the European Regional Development Fund).

## Conflict of Interest

Author VS was employed by company OmniFarma.

The remaining author declares that the research was conducted in the absence of any commercial or financial relationships that could be construed as a potential conflict of interest.

## Publisher’s Note

All claims expressed in this article are solely those of the authors and do not necessarily represent those of their affiliated organizations, or those of the publisher, the editors and the reviewers. Any product that may be evaluated in this article, or claim that may be made by its manufacturer, is not guaranteed or endorsed by the publisher.

## Abbreviations

cTECs, cortical Thymic Epithelial Cells; DCs, Dendritic Cells; DN, Double Negative; DP, Double Positive; ECs, Endothelial Cells; HSCs, Hematopoietic Stem Cells; IL, Interleukin; ILCs, Innate Lymphoid Cells; Fbs, Fibroblasts; LPCs, Lymphoid Progenitor Cells; MFs, Macrophages; MCs, Mesenchymal Cells; MSCs, Mesenchymal Stem Cells; mTECs, medullary Thymic Epithelial Cells; NK, Natural killer; SCF, Stem Cell Factor; SP, Single Positive; Sv, Sievert; TECs, Thymic Epithelial Cells; TEPS, Thymic Epithelial Progenitor Cells; TESCs, Thymic Epithelial Stem Cells; THGF, Thymocyte Growth Factor; TLPs, T Lymphocyte Progenitors; TLSCs, Thymic Lymphoid Stem Cells.
